# A Novel Histological Technique to Assess Severity of Traumatic Brain Injury in Rodents: Comparisons to Neuroimaging and Neurological Outcomes

**DOI:** 10.3389/fnins.2021.733115

**Published:** 2021-10-13

**Authors:** Dmitry Frank, Benjamin F. Gruenbaum, Ilan Shelef, Vladislav Zvenigorodsky, Yair Benjamin, Olha Shapoval, Ron Gal, Alexander Zlotnik, Israel Melamed, Matthew Boyko

**Affiliations:** ^1^Department of Anesthesiology and Critical Care, Soroka University Medical Center, Ben-Gurion University of the Negev, Beer-Sheva, Israel; ^2^Department of Anesthesiology and Perioperative Medicine, Mayo Clinic, Jacksonville, FL, United States; ^3^Department of Radiology, Soroka University Medical Center, Ben-Gurion University of the Negev, Beer-Sheva, Israel; ^4^Department of Physiology, Faculty of Biology, Ecology and Medicine, Dnepropetrovsk State University, Dnepropetrovsk, Ukraine; ^5^Department of Neurosurgery, Soroka University Medical Center, Ben-Gurion University of the Negev, Beer-Sheva, Israel

**Keywords:** histology, magnetic imaging resonance, methods, rodent, traumatic brain injury

## Abstract

Here we evaluate an alternative protocol to histologically examine blood-brain barrier (BBB) breakdown, brain edema, and lesion volume following traumatic brain injury (TBI) in the same set of rodent brain samples. We further compare this novel histological technique to measurements determined by magnetic resonance imaging (MRI) and a neurological severity score (NSS). Sixty-six rats were randomly assigned to a sham-operated, mild TBI, moderate TBI, or severe TBI group. 48 h after TBI, NSS, MRI and histological techniques were performed to measure TBI severity outcome. Both the histological and MRI techniques were able to detect measurements of severity outcome, but histologically determined outcomes were more sensitive. The two most sensitive techniques for determining the degree of injury following TBI were NSS and histologically determined BBB breakdown. Our results demonstrate that BBB breakdown, brain edema, and lesion volume following TBI can be accurately measured by histological evaluation of the same set of brain samples.

## Introduction

Traumatic brain injury (TBI) is a major cause of death and disability in children and young adults (Krug et al., 2000) and has become a critical public health and socio-economic problem globally (Roozenbeek et al., 2013). Traumatic brain injury accounts for one quarter to one third of all accidental deaths, and approximately two thirds of trauma-related deaths in hospitals (Bruns Jr and Hauser, 2003; Corrigan et al., 2010). Many survivors, even those with only minor injuries, suffer from lifelong disability which leads to considerable demands on health services (Langlois and Sattin, 2005). While tremendous efforts have been made in advancing treatment options for both TBI itself and its secondary complications, their efficacy remains far from ideal (Chung and Khan, 2014).

Due to the feasibility and ethical limitations of human studies, animal models serve as well-established alternatives for testing treatment methods and studying the mechanisms and related complications of the condition. Experimental rodent models in particular have historically been the most widely used due to their accessibility, low cost, reproducibility and validated approaches (O’connor et al., 2011; Xiong et al., 2013). Highly precise assessments of parameters of brain tissue destruction and behavioral outcomes are vital to the success of these models. An overview of commonly studied outcomes in experimental rodent models of TBI can be found in Supplementary Material 1. Of these, histological, neuroimaging, behavioral and neurological assessments are the most common techniques used in evaluating TBI in rodent models (Bodnar et al., 2019).

Noninvasive neuroimaging methods have long played an important role in the diagnostic workup and treatment plan following TBI in the clinical setting (Lee and Newberg, 2005). Neuroimaging has also become increasingly used in preclinical experimental models (Hutchinson et al., 2018). Magnetic resonance imaging (MRI), in particular, has been an indispensable tool for assessing the severity of experimental TBI in rats (Fricke et al., 2004; Goetz et al., 2004; Stoffel et al., 2004; Denic et al., 2011). Compared to traditional histological approaches in evaluating brain injury in rodents, MRI avoids euthanasia which is ethically preferred and allows for subsequent behavioral and neurological assessment. However, histological examination and evaluation of motor function are still considered by many as the gold standard for assessing severity of TBI in preclinical rat models (Kobeissy et al., 1940; Osier et al., 2015). In a recent review based on hundreds of published studies of TBI in rats, it was noted that histological evaluations were utilized 55% of the time (Bodnar et al., 2019). The three histological outcomes that especially showed a high sensitivity were blood–brain barrier (BBB) breakdown (94%), brain edema (100%) and assessment of the lesion volume based on axonal injury (95%) or gliosis (93%). Motor skill assays were used 31% of the time (Bodnar et al., 2019). These findings may be due to the economic burden and limited access of availability of MRI equipment in many laboratories.

Another important consideration of preclinical experimental models is the number of animals that are required. A model that requires a large number of animals raises economic and ethical concerns. On the other hand, a small number of animal subjects makes it more difficult to obtain high statistical power in order to ensure reliability and reproducibility. One solution may be to use methods that ensure effective evaluation of a large number of outcomes on the same set of animals without resorting to a larger group size. Obtaining histological measurements of outcomes post-TBI in preclinical experimental models historically requires three separate groups of animals for the evaluation of BBB breakdown, brain edema, and lesion volume, respectively (Başkaya et al., 2000; Meymandi et al., 2018; Soltani et al., 2018). In rodent models of stroke, methods that utilize multiple histological outcomes in the same brain set have been well described (Li et al., 2018; Kuts et al., 2019a; Sanchez Bezanilla et al., 2019). However, no such methods have been previously described in preclinical rodent models of TBI.

The aim of the present study was to apply an alternative protocol to histologically examine BBB breakdown, brain edema, and lesion volume following TBI in the same set of brain samples. For this purpose, we combined the following protocols in a single set of rat brains: measuring brain edema by calculating hemispheric volumes, evaluating BBB breakdown by a spectrometry technique using Evans blue staining, and measuring lesion volume by triphenyl tetrazolium chloride (TTC) staining. To test the sensitivity and efficacy of our new histological method, we further compared the assessment of post-TBI severity in a rodent model utilizing this histological technique to neurological severity outcome and MRI findings, the correlation between which has been considered a gold standard of histological evaluation. This novel approach may serve as a valuable and ethically favorable rodent model of measuring TBI severity.

## Methods

The experiments were conducted in accordance with the recommendations of the Declarations of Helsinki and Tokyo and the Guidelines for the Use of Experimental Animals of the European Community. The experiments were approved by the Animal Care Committee of Ben-Gurion University of the Negev, Israel.

### Animals

The experiments were conducted in a total of 66 male Sprague-Dawley rats (Harlan Laboratories, Israel) weighing between 280 and 320 g each. Purina Chow and water were available *ad libitum*. Rats were maintained in 12:12-h light:dark conditions, at a constant temperature of 22°C ± 1°C. All experiments were conducted in the dark phase, between 08:00 and 16:00.

### Experimental Design

Sixty-six rats were randomly assigned into one of four groups: naïve sham-operated rats (*n* = 15 rats), mild TBI (*n* = 15), moderate TBI (*n* = 16) and severe TBI (*n* = 20). Rats who did not survive the experiment were excluded from the study. The final number of animals in each group was 15 rats (Table 1).

**TABLE 1:** Experimental procedure.

		Group				Total number of rats
			
		Naïve rats	Mild TBI	Moderate TBI	Severe TBI	
**Experimental Procedures**	BBB breakdown assessment	15	15	16 (15 survived)	20 (15 survived)	66
	Lesion volume assessment					
	Brain edema assessment					
	NSS					

*NSS: Neurological severity score. TBI: Traumatic brain injury.*

**FIGURE 1:**
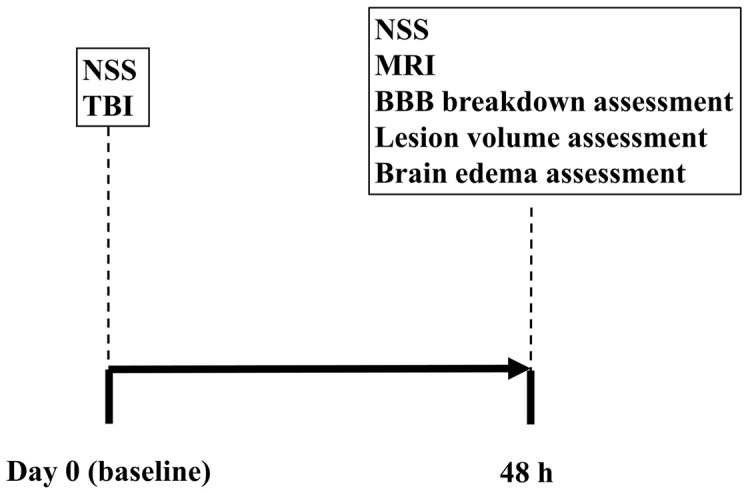
Experimental timeline.

Neurological severity was evaluated before surgery (baseline) and 48 h following various degrees of TBI severity (see Figure 1). After neurological evaluation at 48 h, all rats were scanned on a clinical MRI scanner and subsequently euthanized for histological evaluation of BBB breakdown, brain edema, and lesion volume.

### Neurological Severity Score

Neurological Severity Score (NSS) was determined by two blinded observers, as previously described (Shapira et al., 1988; Boyko et al., 2011a; Ohayon et al., 2012; Zlotnik et al., 2012; Boyko et al., 2013a). Points were assigned for alterations in motor functions and behavior, with the maximum score of 25 representing greatest neurological dysfunction and a score of 0 indicating an intact neurological condition. Specifically, the following were evaluated: ability to exit a circle (3 point scale), gait on a wide surface (3 point scale), gait on a narrow surface (4 point scale), effort to remain on a narrow surface (2 point scale), reflexes (5 point scale), seeking behavior (2 point scale), beam walking (3 point scale), and beam balance (3 point scale).

### Traumatic Brain Injury

Traumatic brain injury (TBI) was performed, as previously described (Jones et al., 2008; Kabadi et al., 2010; Frank et al., 2021). Rats were anesthetized by inhalation of isoflurane (5% for induction and 1.5-2.5% for maintenance) with administration of equal parts medical air and oxygen. The scalp was infiltrated with 0.5% bupivacaine and then incised and reflected laterally with the left temporal muscle, while the underlying periosteum was dissected, exposing the skull. A 5-mm diameter craniotomy was performed with a trephine (Roboz Surgical Instrument Co., Gaithersburg, MD) that attached to a drill bit of an electrical drill (Stoelting, Wood Dale, IL). The center of the craniotomy was positioned 4 mm lateral and 4 mm posterior to bregma. A Luer 3-way stopcock was fixed and additionally held in place by cyanoacrylate adhesive and dental acrylic. Subsequently, the injury was induced by a pressure pulse (mild TBI amplitude of 2.2 atmospheres, moderate TBI amplitude of 2.5 atmospheres, and severe TBI amplitude of 3.0 atmospheres) (Jones et al., 2008; Kabadi et al., 2010). Traumatic brain injury was delivered by a custom-made fluid-percussion device over 21–23 msec through the 3-way stopcock. The fluid pulse from the piston plunger, impacted by the pendulum, was conducted via continuous saline fluid into the dura of the rat, ensuring efficient transmission of the pressure pulse. Sham-operated controls received an identical procedure without the application of the fluid pulse. During the surgical procedure, animals were connected to a pulse-oximeter to enable continuous measurement of heart rate and blood/oxygen levels. Following TBI, the incision was sutured, and the rats were allowed to recover from anesthesia.

### Magnetic Resonance Imaging

Magnetic resonance imaging (MRI) was used for the determination of the volume transfer constant (K^*trans*^) (Liu et al., 2013; Choi et al., 2015), DWI (Boyko et al., 2019b), and T2 (Boyko et al., 2019b) at 48 h following TBI, as described previously (Frank et al., 2019). Animals were maintained under general anesthesia (1.5% isoflurane in oxygen). A tail vein catheter was introduced and connected to a syringe containing a solution of Gadopentetic acid (Gd-DTPA) (Dotarem, 0.5 mmol/ml Guerbet, France). A 3 Tesla (3T) MRI was used (Ingenia, Philips Medical Systems, Best, The Netherlands) using an eight-channel receive-only coil. Localizing T2w turbo spin echo (TSE) sequences were acquired in sagittal and coronal planes with TR/TE = 3000/80 msec, turbo factor = 15, water-fat shift = 1.6 pixels, resolution (freq × phase × slice) = 0.47 × 0.41 × 2.0 mm with a voxel box value size t2 = 0.25×0.25 mm DTI = 0.5 × 0.5 mm, and one average for a scan time of 1:00 min. In the axial direction the scan parameters were repetition time/echo time (TR/TE) = 3000/80 msec, turbo factor = 14, water-fat shift = 1.6 pixels, resolution (freq × phase × slice) = 0.37 × 0.33 × 2.0mm. Four averages were acquired for a scan time of 4:54 min. Diffusion tensor imaging in 6 directions was performed in the axial direction using a multi-shot STimulated Echo Acquisition Mode (STEAM) spin-echo, echo-planar sequence with repetition time/mixing time/echo time (TR/TM/TE) = 1355/15.0/143 msec, SENSitivity Encoding (SENSE) reduction factor = 1.5, turbo factor = 19, b = 1000 s/mm^2^, resolution (freq × phase × slice) = 0.55 × 0.55 × 2.0 mm with spectrally selective fat suppression. Five signal averages were acquired for a scan time of 8:40 min. T1 permeability studies were performed using a segmented 3D T1w-FFE sequence with 50 dynamics for a total scan time of 25:52 min. The scan parameters were TR/TE = 16/4.9 msec, turbo factor = 48, SENSE factor 1.5, resolution (freq × phase × slice) = 0.30 × 0.37 × 2.0 mm, tip angle = 80 and two signal averages for a scan time of 31 sec/dynamic. Three calibration scans with identical resolution preceded the dynamic sequence with tip angles 50, 100, and 150. The contrast agent was injected after the 5th dynamic scan. The Intellispace Portal workstation (V5.0.0.20030, Philips Medical Systems, Best, The Netherlands) was used for the post-processing of the permeability studies.

### Magnetic Resonance Imaging Analysis

Image analysis was performed by an expert, who was blind to the group assignments. Quantitative apparent diffusion coefficient (ADC) maps, in units of square millimeters per second, were generated in Philips software package (Ingenia, Philips Medical Systems, Best, The Netherlands) and subsequently analyzed using ImageJ software (version 1.50i, National Institutes of Health, Bethesda, MD), as previously described (Boyko et al., 2019b). These thresholds were used to identify all pixels of ADC characteristics on each slice. The viability thresholds were 0.53X10-3mm2/s for ADC images (Bardutzky et al., 2005; Boyko et al., 2019b). Calculation of lesion volume was performed by the Ratios of Ipsilateral and Contralateral Cerebral Hemispheres (RICH) method. The calculation of the lesion volume with the correction for tissue swelling by the RICH technique was done using the following formula (Boyko et al., 2013b):


$$\begin{array}{c} Correctedlesionvolume=\\ \frac{Lesionvolume\times Contralateralhemispheresize}{Ipsilateralhemispheresize} \end{array}$$

Calculation of brain edema was also performed by the RICH method (Boyko et al., 2019b). The calculation of brain edema by the RICH technique was done by comparing the contralateral and ipsilateral hemispheres, and performed using the following formula (Boyko et al., 2011a):


$$\begin{array}{c} Brainedema=\\ \frac{Volumeoftherighthemisphere-Volumeofthelefthemisphere}{Volumeofthelefthemisphere} \end{array}$$

The lesion volume and brain edema were measured as a percentage of the total brain (Boyko et al., 2019a).

### Histological Method for Measuring Brain Edema, Lesion Volume, and Blood–Brain Barrier Breakdown

Brain edema, lesion volume, and BBB breakdown were measured in the same set of brain samples following a protocol described previously in a stroke model (Kuts et al., 2019a). 2% Evans blue in saline (4 ml/kg) was administered through a cannulated tail vein as a blood-brain permeability tracer and was allowed to circulate for 60 min. The rats’ chests were opened, and the animals were perfused with cooled saline through the left ventricle. Their brains were quickly isolated and sectioned into 6 coronal slices 2 mm thick. The set of slices from each brain were then incubated for 30 min at 37°C in 0.05% TTC. The slices were scanned with an optical scanner. The images were subsequently used to identify brain edema and lesion volume, as described below. Immediately after scanning, the slices were used to determine the permeability of the BBB, as detailed below.

### Assessment of Brain Edema

We measured the brain edema in pixels, using ImageJ software (Kuts et al., 2019a). Brain edema was expressed as a percentage of the normal areas in the contralateral unaffected hemisphere. Right (injured) cerebral hemisphere edema was determined by calculating the volumes of both hemispheres from the total sum of coronal slice areas. The amount of swelling was calculated using the Kaplan method with this formula: extent of edema = (the volume of right hemisphere – the volume of left hemisphere) / the volume of left hemisphere (Boyko et al., 2019a).

### Assessment of Lesion Volume

We calculated lesion volume in pixels, using the ImageJ software (Kuts et al., 2019a, b), and expressed the result and as a percentage of the normal areas in the contralateral unaffected hemisphere (Boyko et al., 2011a). By numerically integrating six successive 2-mm slices of the marked pale zone, we were able to obtain the total lesion volume. For these measurements, the computer program converts the scan into a black and white image and then uses a threshold function to mask and calculate the pixels that are either black or white (Boyko et al., 2011a). In order to remove the effects of the Evans dye on this process, we added a blue filter using the Channel Mixer function (Image > Adjustments > Channel Mixer) from the Adobe Photoshop CS2 software program prior to calculating brain lesion volume (Kuts et al., 2019a). Lesion volume measurement was performed corrected for tissue edema using the ipsilateral to contralateral cerebral ratio (RICH) method (Boyko et al., 2011b).

### Assessment of Blood–Brain Barrier Disruption

In order to measure BBB disruption, the brain slice samples were weighed and homogenized in trichloroacetic acid, based on the calculation of 1 g of brain tissue in 4 mL of 50% trichloroacetic acid, and was centrifuged at 10,000 × g for 20 min and the supernatant was diluted 1:3 with 96% ethanol. A fluorescence detector was used at an excitation wavelength of 620 nm (bandwidth 10 nm) and an emission wavelength of 680 nm (bandwidth 10 nm) (Frank et al., 2020).

### Statistical Analysis

We used the SPSS 20 package (SPSS Inc., Chicago, IL, United States) for analysis, and addressing the number of rats per group, we applied the Kolmogorov–Smirnov test as a decider of the proper test for comparing between various parameters. With Spearman’s test (for non-parametric data) or Pearson’s test (for parametric data), we calculated the correlation between MRI parameters and histology-obtained results. The significance of comparisons between groups was determined using the Mann–Whitney (for non-parametric data) and by Student’s *t*-tests (for parametric data). Mortality rate was analyzed with chi-square and Fisher’s exact tests. Results were considered statistically significant when *p* < 0.05, and highly significant when *p* < 0.01.

## Results

### Survival Rate

All rats survived in the sham-operated and mild TBI groups. Mortality after 48 h was 6.25% (1 of 16) in the moderate TBI and 25% (5 of 20) in the severe TBI groups. The mortality rate was significant lower in the sham-operated rats compared to the severe TBI group (0% vs. 25%, *p* < 0.05, chi-square and Fisher’s exact test, 1-sided, see Table 1).

### Neurological Severity Score

**FIGURE 2:**
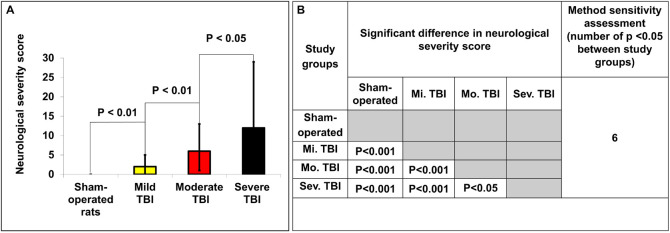
Determination of neurological severity score 48 h after TBI. **(A)** The neurological severity score (NSS) 48 h after TBI in the different experimental groups. There was a significant difference in NSS between groups 48 h after TBI (*p* < 0.05). The data are measured as counts and presented as median ± range. **(B)** Comparison of significant differences (displayed as the p value) between the NSS of different experimental groups.

The NSS for each group 48 h following TBI are presented in Figure 2. As expected, there was no difference between groups at baseline, before intervention. The sham-operated group did not show any neurological deficit at 48 h after TBI (NSS-0). Compared to baseline, the NSS at 48 h was significantly greater after mild [2(2–3) vs. 0(0–0), *U* = 3, *p* < 0.01, *r* = 0.87], moderate [6(5–7) vs. 0(0–0), *U* = 0, *p* < 0.01, *r* = 0.89], and severe [12(5–17) vs. 0(0–0), *U* = 0, *p* < 0.01, *r* = 0.89] TBI, according to the Mann–Whitney test. There was a significant difference in NSS after 48 h between mild, moderate and severe TBI groups (*p* < 0.05). The data are measured as a count and expressed as median and 25–75 percentile range.

### Histological Assessment of Lesion Volume

**FIGURE 3:**
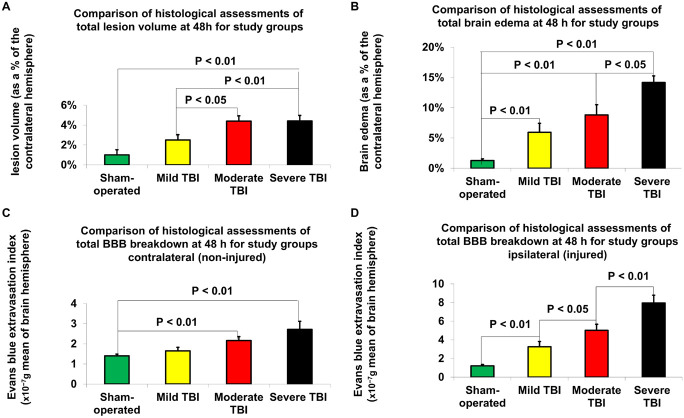
Histological assessment of outcome parameters 48 h after TBI. **(A)** Lesion volume. The data are expressed as a percentage of the contralateral hemisphere and presented as mean ± SEM. **(B)** Brain edema. The data are expressed as a percentage of the contralateral hemisphere and presented as mean ± SEM. **(C)** Blood-brain barrier (BBB) breakdown on contralateral (non-injured) hemisphere. The data are measured in 10^– 7^g of brain tissue and presented as mean ± SEM. **(D)** BBB breakdown on ipsilateral (injured) hemisphere. Significant differences were found between groups of varying injury severity. The data are measured in ng/g of brain tissue and presented as mean ± SEM.

According to Student’s *t*-test, histologically determined lesion volumes 48 h after TBI are presented in Figure 3A. Compared to sham-operated rats (1% ± 0.5%), the lesion volume 48 h after TBI was significantly greater in the moderate [4.4% ± 0.5%, *p* < 0.01, *t*(28) = −4.6, *d* = −1.7] and severe [4.41% ± 0.6%, *t*(28) = −4.5, *p* < 0.01, *d* = −1.6] TBI groups. There was also a significant difference in lesion volume 48 h after TBI between the mild TBI group and the moderate [*t*(28) = −2.5, *p* < 0.05, *d* = 0.9] and severe [*t*(28) = −0.93, *p* < 0.05, *d* = 0.8] TBI groups using the histological technique. The data are expressed as a mean percentage of the contralateral hemisphere ± SEM.

### Histological Assessment of Brain Edema

According to Student’s *t*-test, differences in brain edema, determined by histological examination 48 h after TBI, are presented in Figure 3B. Compared to sham-operated rats (1.3% ± 0.3%), the measurements of brain edema 48 h after TBI were significantly greater in the mild [6% ± 1.5%, *t*(28) = −3.1, *p* < 0.01, *d* = −0.96], moderate [8.8% ± 1.7%, *t*(28) = −4.4, *p* < 0.01, *d* = −1.6] and severe [14.2% ± 1.1%, *t*(28) = −11.4, *p* < 0.01, *d* = −4.11] TBI groups. There was also a significant difference in brain edema 48 h after TBI between the severe TBI group and the mild [*t*(28) = −4.5, *p* < 0.01, *d* = 1.44] and moderate [*t*(28) = −2.7, *p* < 0.05, *d* = 9.98] TBI groups using the histological technique. The data are expressed as a mean percentage of the contralateral hemisphere ± SEM.

### Histological Assessment of Blood–Brain Barrier Breakdown

According to Student’s *t*-test, histologically determined BBB breakdown 48 h after TBI is presented in Figures 3C,D. The BBB breakdown 48 h after TBI was significantly greater for the moderate [2.16 × 10^–7^g ± 0.19 × 10^–7^g, *t*(28) = −3.6, *p* < 0.01, *d* = −1.3] and severe (2.72 × 10^–7^g ± 0.4 × 10^–7^g, *t*(28) = −3.2, *p* < 0.01, *d* = −1.2) TBI groups on the contralateral (non-injured) hemisphere compared to sham-operated rats (1.4 × 10^–7^g ± 0.09 × 10^–7^g (Figure 3C). There was a significant difference in BBB breakdown on the contralateral hemisphere 48 h TBI between the severe and mild TBI groups [t(28) = −2.4, *p* < 0.05, *d* = 0.89]. On the ipsilateral (injured) hemisphere (Figure 3D), compared to sham-operated rats (1.21 × 10^–7^g ± 0.14 × 10^–7^g), the BBB breakdown 48 h after TBI was significantly greater for the mild [3.26 × 10^–7^g ± 0.55 × 10^–7^g, *t*(28) = −3.6, p < 0.01, d = 1.3], moderate [5.02 × 10^–7^g ± 0.65 × 10^–7^g, *t*(28) = −5.8, *p* < 0.01, *d* = 2.1], and severe [7.95 × 10^–7^g ± 0.83 × 10^–7^g, *t*(28) = −8, *p* < 0.01, *d* = 2.9] TBI groups. There was a significant difference between the severe and moderate TBI groups [*t*(28) = −2.8, *p* < 0.01, *d* = 1], severe and mild TBI groups [*t*(28) = −4.7, *p* < 0.01, *d* = 1.7], and mild and moderate TBI groups [*t*(28) = −2.1, *p* < 0.05, *d* = 0.8]. The data are expressed as a mean percentage of the contralateral hemisphere ± SEM.

### Magnetic Resonance Imaging-Determined Lesion Volume

**FIGURE 4:**
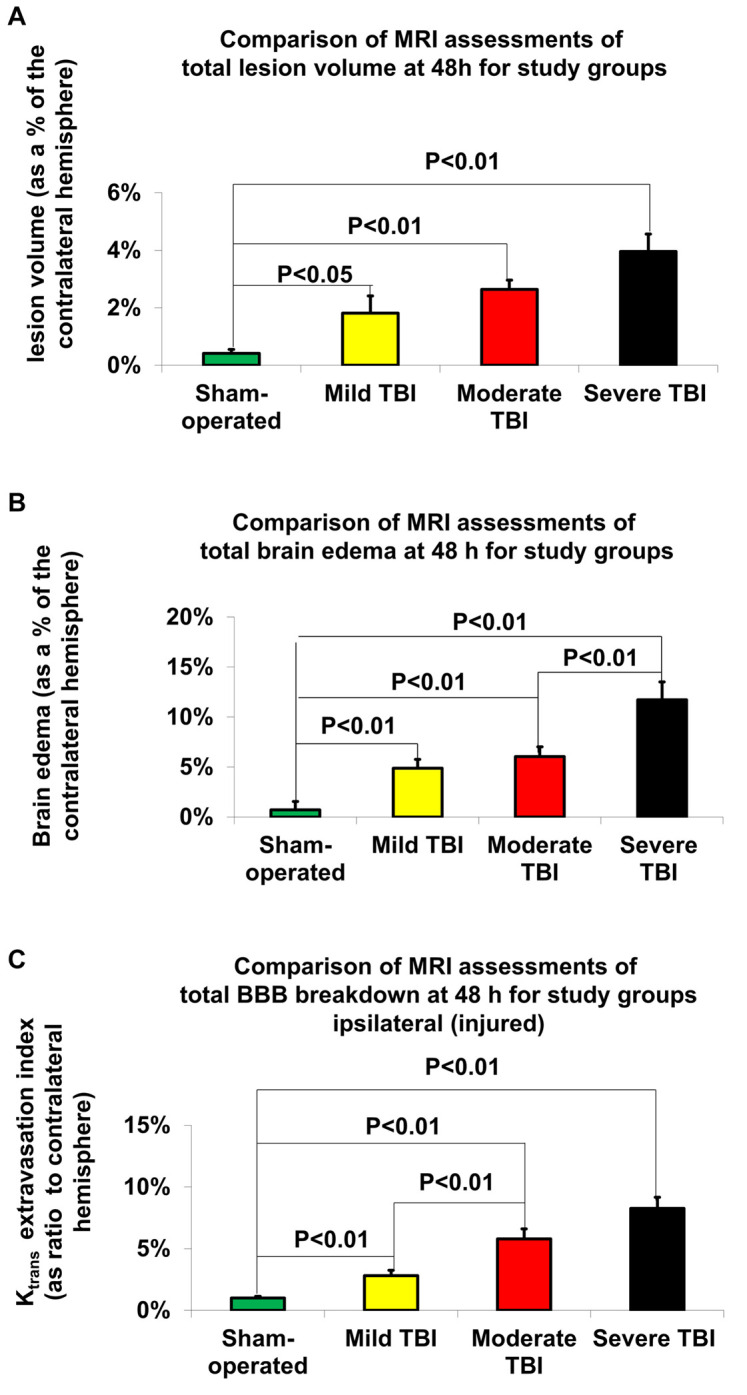
MRI-determined outcome parameters 48 h after TBI. **(A)** Lesion volume. The data are expressed as a percentage of the contralateral hemisphere and presented as mean ± SEM. **(B)** Brain edema. The data are expressed as a percentage of the contralateral hemisphere and presented as mean ± SEM. **(C)** Blood-brain barrier (BBB) breakdown. The data are expressed as a percentage of the contralateral hemisphere and presented as mean ± SEM.

According to Student’s *t*-test, MRI-determined lesion volumes 48 h after TBI are presented in Figure 4A and Table 2A. Compared to sham-operated rats (0.41% ± 0.14%), the lesion volume 48 h after TBI was significantly greater in the mild [1.82% ± 0.6%, *t*(28) = −6.24, *p* < 0.05, *d* = 0.84], moderate [2.63% ± 0.33%, *t*(28) = −6.24, *p* < 0.01, *d* = 2.28], and severe [3.96% ± 0.6%, *t*(28) = −5.7, *p* < 0.01, *d* = 2.07] TBI groups. There was a significant difference in lesion volume 48 after TBI between the mild TBI group and the severe TBI group using the MRI technique [*t*(28) = −2.5, *p* < 0.05, *d* = −0.92]. The data are expressed as a mean percentage of the contralateral hemisphere ± SEM.

**TABLE 2:** Comparison of significant differences (displayed as the p value) between the different experimental groups for (A) lesion volume, (B) brain edema, or (C) blood-brain barrier (BBB) breakdown 48 h after TBI by either histological and MRI-determined techniques.

*Study groups*	Histological				MRI				Method sensitivity assessment (number of *p* < 0.05 between study groups)	
			
	Sham-operated	Mild TBI	Moderate TBI	Severe TBI	Sham-operated	Mild TBI	Moderate TBI	Severe TBI	Histological	MRI
Sham-operated									4	4
Mild TBI	NS				*p* < 0.05					
Moderate TBI	*p* < 0.001	*p* < 0.05			*p* < 0.001	NS				
Severe TBI	*p* < 0.001	*p* < 0.05	NS		*p* < 0.001	*p* < 0.05	NS			

**A**										

	**Histological**				**MRI**				**Method sensitivity assessment (number of *p* < 0.05 between study groups)**	
			
	**Sham-operated**	**Mild TBI**	**Moderate TBI**	**Severe. TBI**	**Sham-operated**	**Mild TBI**	**Moderate TBI**	**Severe TBI**	**Histological**	**MRI**

Sham-operated									5	5
Mild TBI	*p* < 0.01				*p* < 0.01					
Moderate TBI	*p* < 0.001	NS			*p* < 0.001	NS				
Severe TBI	*p* < 0.001	*p* < 0.001	*p* < 0.05		*p* < 0.001	*p* < 0.01	*p* < 0.05			

**B**										

**Study groups**	**Histological**				**MRI**				**Method sensitivity assessment (number of *p* < 0.05 between study groups)**	
			
	**Sham-operated**	**Mild TBI**	**Moderate TBI**	**Severe TBI**	**Sham-operated**	**Mild TBI**	**Moderate TBI**	**Severe TBI**	**Histological**	**MRI**

Sham-operated									6	5
Mild TBI	*p* < 0.01				*p* < 0.001					
Moderate TBI	*p* < 0.001	*p* < 0.05			*p* < 0.001	*p* < 0.01				
Severe TBI	*p* < 0.001	*p* < 0.001	*p* < 0.01		*p* < 0.001	*p* < 0.001	NS			

**C**										

*NS: not significant.*

### Magnetic Resonance Imaging-Determined Brain Edema

According to Student’s *t*-test, differences in brain edema, determined by MRI 48 h after TBI, are presented in Figure 4B and Table 2B. Compared to sham-operated rats (0.72% ± 0.84%), the measured brain edema 48 h after TBI was significantly greater in the mild [4.88% ± 0.88%, *t*(28) = −3.42, *p* < 0.01, *d* = 1.26], moderate [6.05% ± 0.97%, *t*(28) = −4.14, *p* < 0.01, *d* = 1.02], and severe [11.72% ± 1.78%, *t*(28) = −5.59, *p* < 0.01, *d* = 2.24] TBI groups. There was also a significant difference in brain edema 48 h after TBI between the severe TBI group and the mild [*t*(28) = −3.4, *p* < 0.01, *d* = 1.26] and moderate [*t*(28) = −2.8, *p* < 0.01, *d* = 1.02] TBI groups using the MRI technique. The data are expressed as a mean percentage of the contralateral hemisphere ± SEM.

### Magnetic Resonance Imaging-Determined Blood–Brain Barrier Breakdown

According to Student’s *t*-test, MRI-determined BBB breakdown 48 h after TBI is presented in Figure 4C and Table 2C. Using the MRI-technique, compared to the sham-operated rats (1% ± 0.14%), the measured BBB breakdown 48 h after TBI was significantly greater for the mild [2.81% ± 0.45%, *t*(28) = −3.87, *p* < 0.01, *d* = 1.41], moderate [5.79% ± 0.82%, *t*(28) = −5.78, *p* < 0.01, *d* = 2.11] and severe [8.26% ± 0.91%, *t*(28) = −7.91, *p* < 0.01, *d* = 2.88] TBI groups. There was a significant difference in BBB breakdown 48 h after TBI between the mild TBI group and the moderate [*t*(28) = −3.2, *p* < 0.01, *d* = −1.17] and severe [*t*(28) = −5.39, *p* < 0.01, *d* = 1.97] TBI groups using the MRI technique. The data are expressed as a mean percentage of the contralateral hemisphere ± SEM.

### Correlation Comparisons Between the Neurological Severity Score, Magnetic Resonance Imaging and Histological Traumatic Brain Injury Outcomes

**FIGURE 5:**
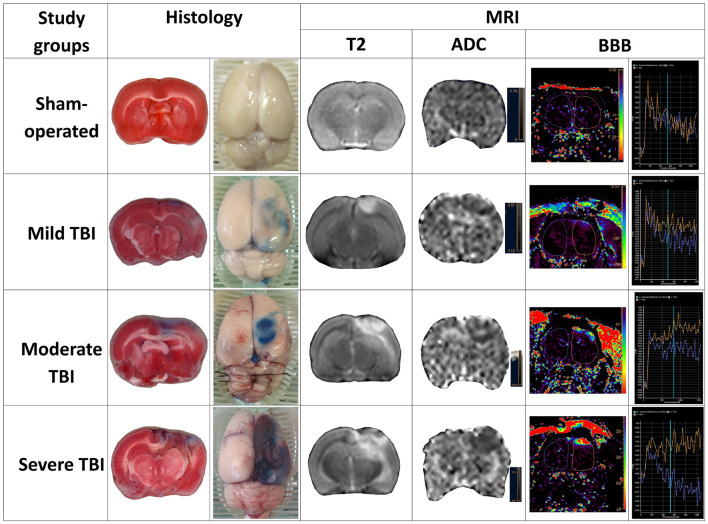
Representative sample of parameters of severity outcome following TBI by histological and MRI-determined techniques in the various experimental groups.

A representative example of each of the various measurements of severity outcome following TBI by histological and MRI-determined techniques is illustrated in Figure 5. Correlations were determined between the different measurements of TBI severity outcome in histological and MRI-determined techniques (see Table 3).

**TABLE 3:** Comparisons between the different measurements of TBI severity outcome. (A) Comparison of the correlation between the different parameters of TBI severity outcome 48 h after TBI by either histological and MRI-determined techniques. (B) Determination of the outcomes with the highest correlation values.

Deferent TBI outcomes		Histological			MRI		
		BBB breakdown	Lesion volume	Brain edema	BBB breakdown	Lesion volume	Brain edema
**Histological**	BBB breakdown						
	Lesion volume	*r*_*p*_ = 0.540 *p* < 0.001					
	Brain edema	*r*_*p*_ = 0.687 *p* < 0.001	*r*_*p*_ = 0.657 *p* < 0.001				
**MRI**	BBB breakdown	*r*_*p*_ = 0.503 *p* < 0.001	*r*_*p*_ = 0.432 *p* < 0.001	*r*_*p*_ = 0.625 *p* < 0.001			
	Lesion volume	*r*_*p*_ = 0.430 *p* < 0.001	*r*_*p*_ = 0.361 *p* < 0.01	*r*_*p*_ = 0.481 *p* < 0.001	*r*_*p*_ = 0.577 *p* < 0.001		
	Brain edema	*r*_*p*_ = 0.537 *p* < 0.001	*r*_*p*_ = 0.478 *p* < 0.001	*r*_*p*_ = 0.757 *p* < 0.001	*r*_*p*_ = 0.539 *p* < 0.001	*r*_*p*_ = 0.337 *p* < 0.01	
**NSS**		*r*_*s*_ = 0.860 *p* < 0.001	*r*_*s*_ = 0.674 *p* < 0.001	*r*_*s*_ = 0.785 *p* < 0.001	r_*s*_ = 0.776 *p* < 0.001	*r*_*s*_ = 0.656 *p* < 0.001	*r*_*s*_ = 0.706 *p* < 0.001

**A**							

			**High**	**Moderate**	**Low**	**Total correlation index**	
	
**NSS**			4	2	0	4.457	

**MRI**		BBB breakdown	1	4	1	3.452	9.648
		Lesion volume	0	2	4	2.842	
		Brain edema	2	2	2	3.354	
**Histological**		BBB breakdown	1	4	1	3.557	10.691
		Lesion volume	0	3	3	3.142	
		Brain edema	2	3	1	3.992	

**B**							

## Discussion

For this experiment, we applied an alternative method to histologically examine BBB breakdown, brain edema, and lesion volume following TBI in the same set of brain samples. We then compared this histological technique to MRI findings and neurological severity outcome following TBI. Our results demonstrate that these parameters of post-TBI neurological injury, BBB breakdown, brain edema and lesion volume, can accurately be measured in the same set of brain samples.

We first examined neurological deficits 48 h after TBI. Neurological regulation has a critical role in mediating motor function, which is governed by a complex neural system beginning in the cortex (Fujimoto et al., 2004). TBI disrupts normal communication between areas of the brain communicating to the spinal cord to produce movement (Fujimoto et al., 2004). Deficits caused by TBI result from disruption of the complex motor pathways and sensorimotor integration. Therefore, most of the tests commonly utilized to assess the outcome of such injury in animal models are sensorimotor (Fujimoto et al., 2004; Xiong et al., 2013).

The NSS and a modified NSS were developed to measure motor function and behavior and are now widely used to assess closed head and unilateral brain injury in rodents (Xiong et al., 2013). In our laboratory we use an updated NSS that is a composite of tests that evaluate motor, sensory, reflex and balance (Ohayon et al., 2012). The neurological assessment method used in our laboratory has been widely validated to detect neurological deficits effectively and sensitively, even in groups of mild TBI rats compared to sham-operated rats (Figures 2A,B). The NSS was significantly different between all groups in our experiment and was the most sensitive test to detect severity after TBI.

We then investigated each of the histological parameters of neurological injury after TBI. We used TTC staining to assess the quantitative destruction of brain tissue, as previously described (Perri et al., 1997; Başkaya et al., 2000). Triphenyl Tetrazolium Chloride staining relies on a process of oxidation, which stains the intact tissues but spares any injured tissues. Triphenyl Tetrazolium Chloride staining has been well-documented as a reliable and sensitive technique for quantitatively determining lesion volume in identifying variations in severity in rodent TBI models (Perri et al., 1997; Başkaya et al., 2000). In fact, TTC staining has been shown to detect damage from even mild TBI (Lim et al., 2017). In contrast, for MRI-detected injury, we used diffusion-weighted imaging (DWI) with ADC mapping. DWI is as a robust and sensitive tool for the determining the area of damage following TBI (Shen et al., 2016) and has been used extensively in detecting many neurologic conditions such as stoke (Saini and Butcher, 2009) and brain tumors (Popp et al., 2019).

Brain edema following TBI is a complex heterogeneous process, associated with an unfavorable prognosis (Jha et al., 2019). In this study, we applied the standardized RICH procedure while calculating the brain edema by histology and MRI, as previously described (Boyko et al., 2019b). The use of this standardized approach in the measurement of brain edema by histological or MRI techniques might explain the high correlation found between these methods (see Table 3A). This is the only outcome that achieved such a high correlation that was not also associated with neurological deficit.

Traumatic brain injury (TBI) causes a disruption in BBB, which assists in regulating healthy brain function (Alves, 2014). Post-TBI, BBB is impacted by a mechanical injury of the microvascular supply, causing disruption of the tight junction complexes, widening of the intercellular spaces, flattening and compression of the vasculature and reduction of the vascular lumen and is followed by cellular swelling (Prakash and Carmichael, 2015; Van Vliet et al., 2020). An immediate BBB breakdown occurs in the early acute phase, with subsequent heightened BBB breakdown for several hours followed by a quick decline. 48 h after brain injury, a second phase of BBB disruption occurs (Belayev et al., 1996). The Evans blue method is a popular, affordable and simple method to histologically evaluate BBB breakdown (Ahishali and Kaya, 2020).

Our innovative protocol studied the effect of the severity of experimental TBI on BBB breakdown. We chose to measure this effect at the peak of the second phase of BBB destruction 48 h after injury, as previously described (Belayev et al., 1996). Measurement of BBB breakdown in the injured hemisphere showed a significant increase in relation to TBI severity (Figure 3D and Table 2C). In addition, there was a significant change in BBB breakdown recorded in the healthy hemisphere following moderate or severe TBI (Figure 3C). This method, like the neurological outcome, showed a very high sensitivity in detecting TBI severity.

In the setting of TBI, injury-related BBB breakdown aggravates brain edema, which critically impacts clinical outcome (Jha et al., 2019). This relationship between BBB breakdown and brain edema may be a reason why these two parameters were so highly correlated. While previous studies have described the varying degrees of brain edema in relation severity following TBI in rodent models (Ren and Lu, 2019), this is the first study to our knowledge that clearly demonstrates the efficacy of histologically measuring BBB breakdown to determine TBI severity.

Our study found that the two most sensitive outcomes in our new histological technique for determining the degree of injury following TBI were NSS and BBB breakdown. Both outcomes showed significant differences between all variations of TBI severity (Table 2C and Figure 3D for BBB breakdown and Figures 2A,B for NSS). The next most sensitive outcomes in TBI were (1) BBB as measured by an MRI technique (Figure 4C and Table 2C); (2) brain edema as measured by our new histological technique (Figure 3B and Table 2B); and (3) brain edema as measured by MRI (Figure 4B and Table 2B). The least sensitive parameter was lesion volume measured either by MRI or the histological technique (Figures 3A, 4A and Table 2A).

Overall, it should be noted that the highest value of correlation, calculated as the sum of all correlations of neurological deficit with other outcomes, was noted in the NSS (Tables 3A,B). The NSS was highly correlated with 4 different methods compared with only 2 high correlations associated with MRI and histological methods (Table 3B). Moreover, unlike MRI and histological techniques, there were no low correlations found between NSS and other methods (Table 3B). The explanation for this phenomenon is that a combination of all damage factors, including brain edema, lesion volume and BBB breakdown, affects neurological deficit. Neurological deficit appears to be the most effective and sensitive test, reflecting the severity of TBI across these areas.

In general, all histological outcomes showed a greater correlation in comparison with MRI outcomes (Total correlation index *r* = 10.691 for histological outcomes versus *r* = 9.648 for MRI outcomes, in Tables 3A,B). We associate this with the technical capabilities of the equipment. Magnetic Resonance Imaging images have yet to reach the resolution and quality obtained with the histological technique (Bridge and Clare, 2006), which explains why histological staining is considered a more accurate technique. Thus, these findings emphasize why histologic evaluation remains the gold standard for evaluating outcomes, and MRI, despite its utility for *in vivo* imaging, is an inferior estimation (Bodnar et al., 2019).

In addition to assessing the correlation between MRI and the histological findings, we determined the sample size of experimental groups (TBI group versus sham). Calculations were based on an alpha set at 0.05, two-tailed; power 80%; mean and standard deviation (see Supplementary Table 1). Since the general trend indicated higher correlations associated with the histological outcomes, we expected to find a similar trend in this statistical method, with a smaller group size in histological outcomes compared to MRI. We were surprised to find that the trend indicated a smaller sample size in MRI groups (especially in lesion volume and brain edema outcomes), compared to histological outcomes. We associate this with the mutually reinforcing influence between the ADC and T2 dimensions. As noted above, it is believed that the best MRI outcome to correlate with the histological determination of the brain lesion volume is the ADC measurement. However, studies show that cytotoxic edema also has a significant impact on the ADC measurement (Ito et al., 1996; Putten et al., 2005). The T2 measurement used to measure cerebral edema can be influenced by the extent of the lesion volume (Milidonis et al., 2015). Thus, it is logical to assume that both ADC and T2 are related (Loubinoux et al., 1997) and mutually reinforce each other.

As expected, we did not find a strong correlation between the lesion volume measured by histology and MRI-detected lesion volumes. The assessment of the lesion volume in the histological method of TTC staining and with the MRI techniques are based on different principles. While TTC staining relies on a process of oxidation, the MRI technique assesses the quantitative destruction of brain tissue using diffusion. In addition, as previously shown, cytotoxic cerebral edema can influence the MRI technique (Saini and Butcher, 2009), but this does not occur with TTC staining (Loubinoux et al., 1997). Similarly, BBB breakdown measured using MRI and histological techniques showed only a moderate correlation. This is likely due to the mechanistic discrepancies between MRI and histology in BBB breakdown measurements that also cause a low correlation between the two measurements of lesion volume.

The validity of the measurement of brain edema by both histological and MRI techniques is suggested by the high correlation between them (Table 3A). This may reflect several factors. First, the resolution of T2 images that calculate brain edema is much higher than the resolution of the images obtained when using the diffusion algorithm by MRI. Second, as noted above, the calculation of brain edema in both techniques was performed by the RICH method (Boyko et al., 2019b), which compares the contralateral and ipsilateral hemispheres. The difference between these two techniques, however, is that the images in the histological method were obtained with a scanner using a dissected rat brain, while the MRI images were obtained from the brain of a living rat. However, the boundary of the brain tissue was clearly defined by the computer program in both cases.

There were several limitations to this experiment. First, it is not standard practice to use a threshold to adjust the ADC map measurement. However, we believe that this important parameter affects the accuracy of the measurement (Boyko et al., 2019b; Kuts et al., 2019a) and therefore was used in our protocol. It should also be mentioned that this parameter is influenced by many factors, including the size of the brain (Yushmanov et al., 2002; Purushotham et al., 2015), brain region (Yuan et al., 2017), model of the disease under study (Bardutzky et al., 2005; Albrecht et al., 2019) and age-related trends in the rat (Bardutzky et al., 2005; Bontempi et al., 2021). We used the recommended optimal threshold parameters for correction of ADC maps (Benveniste et al., 1992; Yushmanov et al., 2002; Meng et al., 2004; Bra et al., 2009), which have been tested in rat ischemia models (Boyko et al., 2019b). We recognize that when using different animals, different types of rats, rats of different ages, or performing analysis of other brain regions using different MRI machines or other software, the parameters of the threshold for ADC may differ slightly.

Another limitation of this study may involve the use of a human 3T MRI while many animal studies use high-field animal MRI, which would have better resolution and sensitivity. However, we have shown quantitative and qualitative similarities in determining brain injury in rodents with a 3T MRI compared to higher Tesla magnets (Boyko et al., 2019b). In addition, we only used male rats in this study. To our knowledge, there have not been any documented differences in the literature between male or female rats in histological staining techniques. We utilized only male rats in order to minimize variability in severity of TBI injury from gender differences (Zlotnik et al., 2011; Tsesis et al., 2013).

In conclusion, we demonstrated that our new histological protocol for evaluating three outcomes on one set of brains was effective in assessing lesion volume, brain edema and BBB breakdown in TBI groups compared to sham-operated rats. Our new protocol was sensitive enough to find significant differences between groups of varying severity of TBI, and in general, these histologically determined outcomes were more sensitive than the MRI-detected outcomes. Each of the parameters analyzed in this study, including motor function and behavior, brain edema, lesion volume, and BBB, should be examined in combination to quantitatively assess the amount of neurological damage. We expect that this protocol can be easily reproduced and applied as an alternative to or in conjunction with MRI, NSS, and other measurements of brain damage following TBI.

## Data Availability Statement

The raw data supporting the conclusions of this article will be made available by the authors, without undue reservation.

## Ethics Statement

The animal study was reviewed and approved by Animal Care Committee of Ben-Gurion University of the Negev, Israel.

## Author Contributions

DF and MB: study conception, data collection, data analysis, manuscript writing/editing, and final approval of the manuscript. BG: data collection, data analysis, the manuscript writing/editing, and final approval of the manuscript. IS, VZ, YB, OS, RG, AZ, and IM: data collection, data analysis, the manuscript editing, and final approval of the manuscript. All authors contributed to the article and approved the submitted version.

## Conflict of Interest

The authors declare that the research was conducted in the absence of any commercial or financial relationships that could be construed as a potential conflict of interest.

## Publisher’s Note

All claims expressed in this article are solely those of the authors and do not necessarily represent those of their affiliated organizations, or those of the publisher, the editors and the reviewers. Any product that may be evaluated in this article, or claim that may be made by its manufacturer, is not guaranteed or endorsed by the publisher.
