# Supplementary material for: A Novel Histological Technique to Assess Severity of Traumatic Brain Injury in Rodents: Comparisons to Neuroimaging and Neurological Outcomes
Source: Front Neurosci. 2021 Oct 13;15:733115. doi: 10.3389/fnins.2021.733115 (PMC8549653; doi:10.3389/fnins.2021.733115)
Supplement: Supplementary file 1 [file Data_Sheet_1.DOCX]

| **Different outcomes** | | | |
| --- | --- | --- | --- |
| Histological outcome | Brain edema^1^ | | |
|  | Blood-brain barrier damage^2^ | | |
|  | Quantitative assessment of brain tissue damage | Cell or tissue changes | Hematoxylin and eosin^2^ |
|  |  |  | TTC^3^ |
|  |  |  | Cresyl violet^4^ |
|  |  |  | Nissl staining^5^ |
|  |  | Proteins of interest | Axonal injury shown by amyloid precursor protein,^6^ silver staining,^7^ neurofilament^8^ |
|  |  |  | Gliosis shown by glial fibrillary acidic protein, ionized calcium binding adaptor molecule 1, CB68, or CD11b staining^9, 10^ |
|  |  |  | Myelin changes^11^ |
|  |  |  | Western blot analysis^12^ |
|  |  |  | GFAP^13^ |
|  |  |  | labeling or apoptosis staining^13^ |
|  |  |  | inflammation/immune activation^14^ |
|  |  |  | Cell-free DNA^1^ |
|  |  |  | Caspase activation^13^ |
|  |  |  | Activation of microglia^13^ |
|  |  |  | Complement activation^15^ |
| **Motor function^16^** | | | |
| Neuro-Visualization | MRI^17^ | | |
|  | MRS^18^ | | |
|  | PET imaging^19^ | | |
| **Blood biomarkers^20^** | | | |
| **Genetics^21^** | | | |
| Behavioral assessments^16, 22^ | Depression^22^ | | |
|  | Anxiety^22^ | | |
|  | Irritability^22^ | | |
|  | Aggression^22^ | | |
|  | Social behavioral^23^ | | |
|  | Cognition^24^ | | |
|  | Memory^25^ | | |

**Supplement 1. Overview of common outcomes studied in the experimental rodent TBI model.** DNA: deoxyribonucleic acid. GFAP: Glial fibrillary acidic protein. MRI: Magnetic resonance imaging. MRS: Magnetic resonance spectroscopy. PET: Positron emission tomography. TTC: 2,3,5-Triphenyltetrazolium chloride.

**References**:

1. Ohayon, S.*, et al.* Cell-free DNA as a marker for prediction of brain damage in traumatic brain injury in rats. *J Neurotrauma* **29**, 261-267 (2012).

2. Song, Y.M.*, et al.* Differences in pathological changes between two rat models of severe traumatic brain injury. *Neural Regen Res* **14**, 1796-1804 (2019).

3. Başkaya, M.K., DOĞAN, A., Temiz, C. & Dempsey, R.J. Application of 2, 3, 5-triphenyltetrazolium chloride staining to evaluate injury volume after controlled cortical impact brain injury: role of brain edema in evolution of injury volume. *Journal of neurotrauma* **17**, 93-99 (2000).

4. Jia, F.*, et al.* MMP-9 inhibitor SB-3CT attenuates behavioral impairments and hippocampal loss after traumatic brain injury in rat. *Journal of neurotrauma* **31**, 1225-1234 (2014).

5. Chen, X.*, et al.* Omega-3 polyunsaturated fatty acid attenuates traumatic brain injury-induced neuronal apoptosis by inducing autophagy through the upregulation of SIRT1-mediated deacetylation of Beclin-1. *Journal of Neuroinflammation* **15**, 1-15 (2018).

6. Zhang, P.*, et al.* Quantitative proteomics analysis to identify diffuse axonal injury biomarkers in rats using iTRAQ coupled LC–MS/MS. *Journal of proteomics* **133**, 93-99 (2016).

7. Hall, E.D., Bryant, Y.D., Cho, W. & Sullivan, P.G. Evolution of post-traumatic neurodegeneration after controlled cortical impact traumatic brain injury in mice and rats as assessed by the de Olmos silver and fluorojade staining methods. *Journal of neurotrauma* **25**, 235-247 (2008).

8. Posmantur, R., Newcomb, J., Kampfl, A. & Hayes, R. Light and confocal microscopic studies of evolutionary changes in neurofilament proteins following cortical impact injury in the rat. *Experimental neurology* **161**, 15-26 (2000).

9. Clark, D.P.*, et al.* Inflammation in traumatic brain injury: roles for toxic A1 astrocytes and microglial–astrocytic crosstalk. *Neurochemical research* **44**, 1410-1424 (2019).

10. Das, M.*, et al.* Lateral fluid percussion injury of the brain induces CCL20 inflammatory chemokine expression in rats. *Journal of neuroinflammation* **8**, 1-16 (2011).

11. Glushakov, A.O.*, et al.* Chronic upregulation of cleaved-caspase-3 associated with chronic myelin pathology and microvascular reorganization in the thalamus after traumatic brain injury in rats. *International journal of molecular sciences* **19**, 3151 (2018).

12. Feng, Y.*, et al.* Neuroprotective effects of resveratrol against traumatic brain injury in rats: involvement of synaptic proteins and neuronal autophagy. *Molecular medicine reports* **13**, 5248-5254 (2016).

13. Tang, W.-C.*, et al.* Early electroacupuncture treatment ameliorates neuroinflammation in rats with traumatic brain injury. *BMC complementary and alternative medicine* **16**, 470 (2016).

14. Schober, M.E.*, et al.* Docosahexaenoic acid decreased neuroinflammation in rat pups after controlled cortical impact. *Experimental neurology* **320**, 112971 (2019).

15. Bellander, B.-M., Bendel, O., von Euler, G., Ohlsson, M. & Svensson, M. Activation of microglial cells and complement following traumatic injury in rat entorhinal-hippocampal slice cultures. *Journal of neurotrauma* **21**, 605-615 (2004).

16. Shultz, S.R.*, et al.* The clinical relevance of behavior testing in animal models of traumatic brain injury. *Journal of Neurotrauma*  (2019).

17. Shen, Q., Watts, L.T., Li, W. & Duong, T.Q. Magnetic resonance imaging in experimental traumatic brain injury. in *Injury Models of the Central Nervous System* 645-658 (Springer, 2016).

18. Singh, K.*, et al.* Altered metabolites of the rat hippocampus after mild and moderate traumatic brain injury–a combined in vivo and in vitro 1H–MRS study. *NMR in Biomedicine* **30**, e3764 (2017).

19. Wang, Y.*, et al.* PET imaging of neuroinflammation in a rat traumatic brain injury model with radiolabeled TSPO ligand DPA-714. *European journal of nuclear medicine and molecular imaging* **41**, 1440-1449 (2014).

20. Jaber, Z.*, et al.* Role of systems biology in brain injury biomarker discovery: neuroproteomics application. in *Injury Models of the Central Nervous System* 157-174 (Springer, 2016).

21. Pan, Y.-B., Sun, Z.-L. & Feng, D.-F. The role of microRNA in traumatic brain injury. *Neuroscience* **367**, 189-199 (2017).

22. Malkesman, O., Tucker, L.B., Ozl, J. & McCabe, J.T. Traumatic brain injury–modeling neuropsychiatric symptoms in rodents. *Frontiers in neurology* **4**, 157 (2013).

23. Mychasiuk, R., Hehar, H., Farran, A. & Esser, M.J. Mean girls: sex differences in the effects of mild traumatic brain injury on the social dynamics of juvenile rat play behaviour. *Behavioural brain research* **259**, 284-291 (2014).

24. Titus, D.J.*, et al.* Positive allosteric modulation of the α7 nicotinic acetylcholine receptor as a treatment for cognitive deficits after traumatic brain injury. *PloS one* **14**, e0223180 (2019).

25. McGuire, J.L.*, et al.* Pioglitazone improves working memory performance when administered in chronic TBI. *Neurobiology of Disease* **132**, 104611 (2019).
