# Supplementary material for: A Novel Histological Technique to Assess Severity of Traumatic Brain Injury in Rodents: Comparisons to Neuroimaging and Neurological Outcomes
Source: Front Neurosci. 2021 Oct 13;15:733115. doi: 10.3389/fnins.2021.733115 (PMC8549653; doi:10.3389/fnins.2021.733115)
Supplement: Supplementary file 2 [file Table_1.DOCX]

| ***Study groups*** | ***Histological*** | | | ***MRI*** | | | ***NSS*** |
| --- | --- | --- | --- | --- | --- | --- | --- |
|  | IZ | BE | BBB | ADC | T2 | K_trans_ |  |
| Mild TBI | ***n=22; 2.5%±2.05% vr. 1%±2%*** | ***n=26; 6.0%±5.8% vr. 1.3%±1.1%*** | ***n=12;***  ***3.3***x10−^7^g***±2.1***x10^−7^g ***vr. 1.2***x10−^7^g***±0.5***x10^−7^g | ***n=6;***  ***1.8%±2.3% vr. 0.4%±0.5%*** | ***n=8; 4.9%±3.4 vr. 0.7%±3.3%*** | ***n=8; 2.8%±1.7 vr. 1%±0.5%*** | ***n=2; 2.48±1.06 vr. 0.13±0.35*** |
| Moderate TBI | ***n=8; 4.4%±2.07% vr. 1%±2%*** | ***n=16; 8.8%±6.5% vr. 1.3%±1.1%*** | ***n=6;***  ***5.0***x10−^7^g***±5.5***x10^−7^g ***vr. 1.2***x10−^7^g***±0.5***x10^−7^g | ***n=2;***  ***2.6%±1.3 vr. 0.4%±0.5%*** | ***n=6; 6.1%±3.8 vr. 0.7%±3.3%*** | ***n=6; 5.8%±3.2 vr. 1%±0.5%*** | ***n=2; 5.73±1.03 vr. 0.13±0.35*** |
| Severe TBI | ***n=8; 4.42%±2.16% vr. 1%±2%*** | ***n=4; 14.2%±4.3% vr. 1.3%±1.1%*** | ***n=4;***  ***7.0***x10−^7^g***±3.2***x10^−7^g ***vr. 1.2***x10−^7^g***±0.5***x10^−7^g | ***n=2;***  ***4%±2.3 vr. 0.4%±0.5*** | ***n=4; 11.7%±6.9 vr. 0.7%±3.3%*** | ***n=4; 8.3%±3.5 vr. 1%±0.5%*** | ***n=2; 12.53±7.4 vr. 0.13±0.35*** |
| **Table 1** | | | | | | | |

**Table 1. Method sensitivity assessment.** We determined the sample size of experimental groups (TBI group versus sham). The data are expressed as a mean ± SD. n is the total number of cases in 2 equivalent groups.
